# Salient networks: a novel application to study Alzheimer disease

**DOI:** 10.1186/s12938-018-0566-5

**Published:** 2018-11-20

**Authors:** Nicola Amoroso, Domenico Diacono, Marianna La Rocca, Roberto Bellotti, Sabina Tangaro, The Alzheimer’s Disease Neuroimaging Initiative

**Affiliations:** 1 Dipartimento Interateneo di Fisica “M. Merlin”, Università degli Studi di Bari “A. Moro”, Via Giovanni Amendola 173, 70125 Bari, Italy; 2 Istituto Nazionale di Fisica Nucleare, Sezione di Bari, Via Orabona 4, 70123 Bari, Italy

**Keywords:** Salient network, Alzheimer’s disease, Scale-free, Small-world, MCI

## Abstract

**Background:**

Extracting fundamental information from data, thus underlining hidden structures or removing noisy information, is one of the most important aims in different scientific fields especially in biological and medical sciences. In this article, we propose an innovative complex network application able to identify salient links for detecting the effect of Alzheimer’s disease on brain connectivity. We first build a network model of brain connectivity from structural Magnetic Resonance Imaging (MRI) data, then we study salient networks retrieved from the original ones.

**Results:**

Investigating informative power of the salient skeleton features in combination with those of the original networks we obtain an accuracy of $0.91 \pm 0.01$ for the distinction of Alzheimer disease (AD) patients from normal controls (NC). This performance significantly overcomes accuracy of the original network features. Moreover salient networks are able to correctly discriminate normal controls (NC) from AD patients and NC from subjects with mild cognitive impairment that will convert to AD (cMCI). These evaluations, performed on an independent dataset, give an accuracy of $0.79 \pm 0.01$ and $0.76 \pm 0.01$ respectively for NC-AD and NC-cMCI classifications. Therefore, most of the informative content of the original networks is kept after the 92 $\%$ and 82 $\%$ reduction respectively in the number of nodes and links. In addition, the present approach, applied to a publicly available MRI dataset from the Alzheimer Disease Neuroimaging Initiative (ADNI), brings out also some interesting aspects related to the topologies and hubs of the networks.

**Conclusions:**

The experimental results demonstrate how salient networks can highlight important brain network characteristics and structural pathological changes, while reducing considerably data complexity and computational requirements.

## Background

Alzheimer’s disease (AD) is a progressive neurodegenerative disease accounting for most cases of dementia after the age of 65. AD onset is related to cognitive impairment and loss of memory and it is estimated that over 115 million people will develop AD by 2050 [1, 2].

AD related brain changes can be detected in vivo with magnetic resonance imaging (MRI) that has been playing an increasingly important role for the early diagnosis of neurodegenerative disorders. Progression of AD can begin even decades before the clinical and radiological manifestations of the illness. Indeed, it is known that dementia is preceded by a prodromal phase of mild cognitive impairment [3], and this, in turn, by a pre-clinical phase [4] of variable duration. Understanding the biological changes, occurring in these early phases, is of fundamental importance for the production of future disease-modifying treatments. Recently, cerebrospinal fluid analyses and brain imaging using radioactive tracers can tell us to what extent the brain is covered with plaques and tangles. However, these methods are very invasive, expensive and only available at some specific centers.

In fact, standard imaging techniques with structural MRI have allowed the observation of gray matter reductions especially in the hippocampus, the enthorinal cortex and the para-hippocampal gyrus in both temporal lobes [5–7]. As a result, great effort has been given to the implementation of fully automated whole brain [8, 9] and ROI [10, 11] segmentation strategies to obtain a robust base of knowledge for developing supervised models. Nonetheless, recent works [12, 13] have disclosed that when considering data from multi-center databases, structural features provided by segmentation approaches and the supervised algorithms based on them to distinguish normal controls and Alzheimer disease subjects are hardly able to reproduce the performances reached on a same type of data. Besides, the processing of numerous imaging databases along with the intrinsic high dimensionality of each brain scan composed of $10^6$ voxel per scan makes the research of novel data mining and managing methods of strategical importance to extract meaningful information aimed at an early diagnosis of AD.

Machine learning techniques remain unsurpassed in terms of accuracy and yet these strategies seem the best chance to get an early diagnosis for AD. However, complex networks, can be a convenient and innovative instrument to describe the connectivity of both structural and functional brain networks and detect anomalies yielded by disease [14, 15] lessening the role played by ROI detection that, on the contrary, needs a preliminary segmentation introducing inevitably a bias and does not allow the discovery of new regions connected to the disease.

Numerous studies, across a different range of anatomical parts of the brain, scales and modalities have found that networks may show a behavior outcome of a combination of both regularity and randomness [16]. In fact, it is known that brain networks can exhibit, at multiple levels, both small-world and scale-free properties in order to optimize brain organization and robustness respectively [17]. A network is called scale-free when degree distribution follows a power law distribution. Therefore, scale-free networks present, most of the nodes, with a limited number of connections and a small number of nodes, called hubs, with a large number of connections. A network is considered small-world if it is highly clustered locally and has a small separation globally, these two tendencies are measured respectively by clustering coefficient and average path length.

Currently, many studies emphasized as scale-free and small-world topologies might be used to model human brain networks and as a consequence describe how brain connectivity is affected by AD. In fact, it was shown that several diseases, such as Alzheimer’s disease, disrupt small-world organization and make human brains more adherent to random or regular networks. However, nothing definitive has been established, yet. Besides, another interesting aspect reported in literature is the vulnerability of network hubs to targeted attacks, these latters often are related to the progression of the disease in the network [18].

In this study, for each MRI T1 brain scans, we modeled brain connectivity dividing automatically brain into a fixed number of boxes, called patches or supervoxels, to determine the nodes of the network and measuring pairwise Pearson’s correlation for all supervoxels to define the network links. Thus, a weighted and undirected dense network was built for each subject scans. One of the major aims of this work is the identification of hubs or highways, which could be modified by the disease. Therefore, it was carried out a study to define a proper threshold to bring out the two main topologies of the brain networks of which peculiar elements are really the most connected nodes and the most important links. [19, 20]. At this point to focus on these elements, we analyzed how a network based on salient links allows successfully prediction of the behavior of different clinical groups [21]. The informative contribute of the salient skeleton was assessed with the multiplex network approach [22] in order to extract several network features for feeding a supervised classification model and detecting AD patterns.

The paper includes: a section of "Methods" where we provide a description of the data used and an overview of the image processing pipeline. Then, we illustrate the modeling of the networks and the characterization of their topology. Finally, we show the salience skeleton construction and hubs detection and the classification phase. In "Results" section we present our findings and in the "Discussion" and Conclusions sections we report respectively the result interpretation and the work summarization along with the future perspectives.

## Methods

**Fig. 1:**
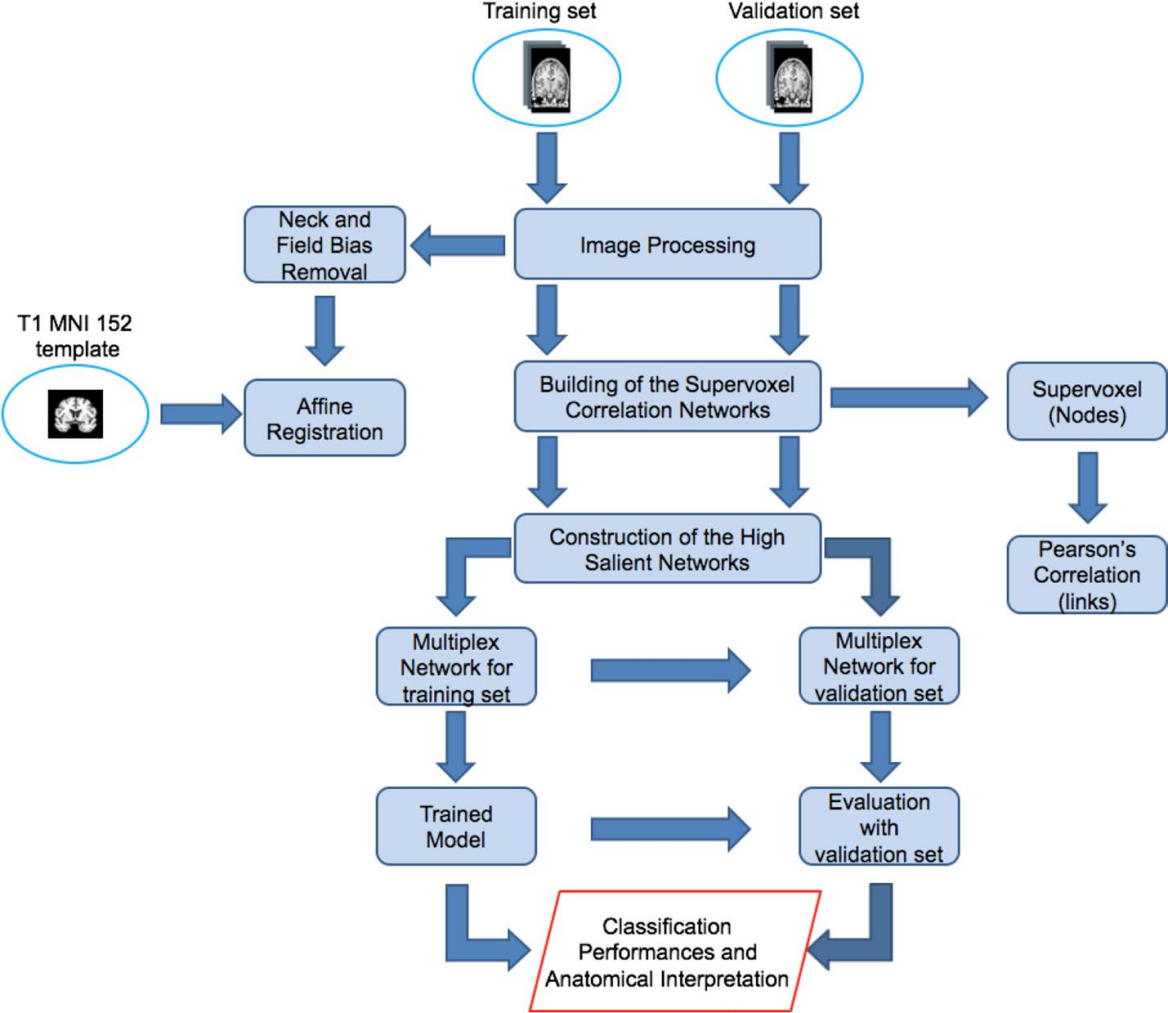
The whole analysis pipeline. The figure shows a schematic overview of the proposed methodology which encompasses different phases: image normalization, brain network model, high salient skeleton construction and supervised learning for the method evaluation

The proposed approach includes four main steps shown in Fig. 1:

- Image processing to achieve an intensity and spatial normalization among subjects;
- Network construction for each subject having supervoxels as nodes and their Pearson’s correlations as links;
- Detection of the salience links to retrieve the salient skeleton from the original network;
- Construction of the multiplex network of the salient skeletons to extract some features in order to assess informative power of the skeletons using supervised learning techniques.

### Image pre-processing and brain network model

In this work we used a training set of 67 T1 MRI scans, composed of 29 normal controls (NC) and 38 AD subjects, from the Alzheimer’s Disease Neuroimaging Initiative (ADNI). These subjects belonged to a larger benchmark dataset [23] selected in order to obtain a compact yet representative sample of ADNI. We also employed an independent validation set of 148 subjects, composed by 52 NC, 48 AD and 48 subjects with mild cognitive impairment converting to AD (cMCI). Validation set subjects were randomly chosen within the whole ADNI in order to match the demographic characteristics of training subjects. Demographic characteristics are reported in the following Table 1. Clinical status, population size, age (average and standard deviation) and gender are provided.

**Table 1:** This table reports the clinical and demographic information of the sets employed in this study

	Training set		Validation set			Total
Disease status	AD (38)	NC (29)	AD (48)	NC (52)	cMCI (48)	215
Female/male	18/20	13/16	22/26	25/27	21/27	99/116
Age (years)	$74\ \pm \ 8$	$75\ \pm \ 6$	$78\ \pm \ 6$	$75\ \pm \ 6$	$76\ \pm \ 6$	$76\ \pm \ 6$
MMSE	$23\ \pm \ 2$	$29\ \pm \ 1$	$24\ \pm \ 2$	$29\ \pm \ 1$	$27\ \pm \ 2$	$26\ \pm \ 2$

Data size, age range, gender and Mini Mental State Examination (MMSE) are shown for each diagnostic group with the relative mean and standard deviation

Firstly, MRI scans were intensity normalized to reduce inter-subject differences. Then, FSL-BET and FSL-FLIRT, from FMRIB library [9], were applied to the images for brain identification and for carrying out a spatial affine registration on the MNI152 T1 template. To avoid any bias due to pre-processing, all these steps were performed with standard configurations and parameters. After brain scans had been normalized, both in intensity and spatially, we divided, for each subject, the whole brain into $N=549$ box called supervoxels of volume of 3000 mm^3^, without a preliminary segmentation. As shown in previous works [24–26], the use of this supervoxel size is connected to the characteristic size of anatomical brain regions affected by AD, such as the hippocampus. The pairwise similarity of supervoxels was computed by means of Pearson’s correlation, therefore an undirected weighted network, of which nodes are the supervoxels and links are the correlation measurements, was constructed for each scan.

### High salient skeleton construction

**Fig. 2:**
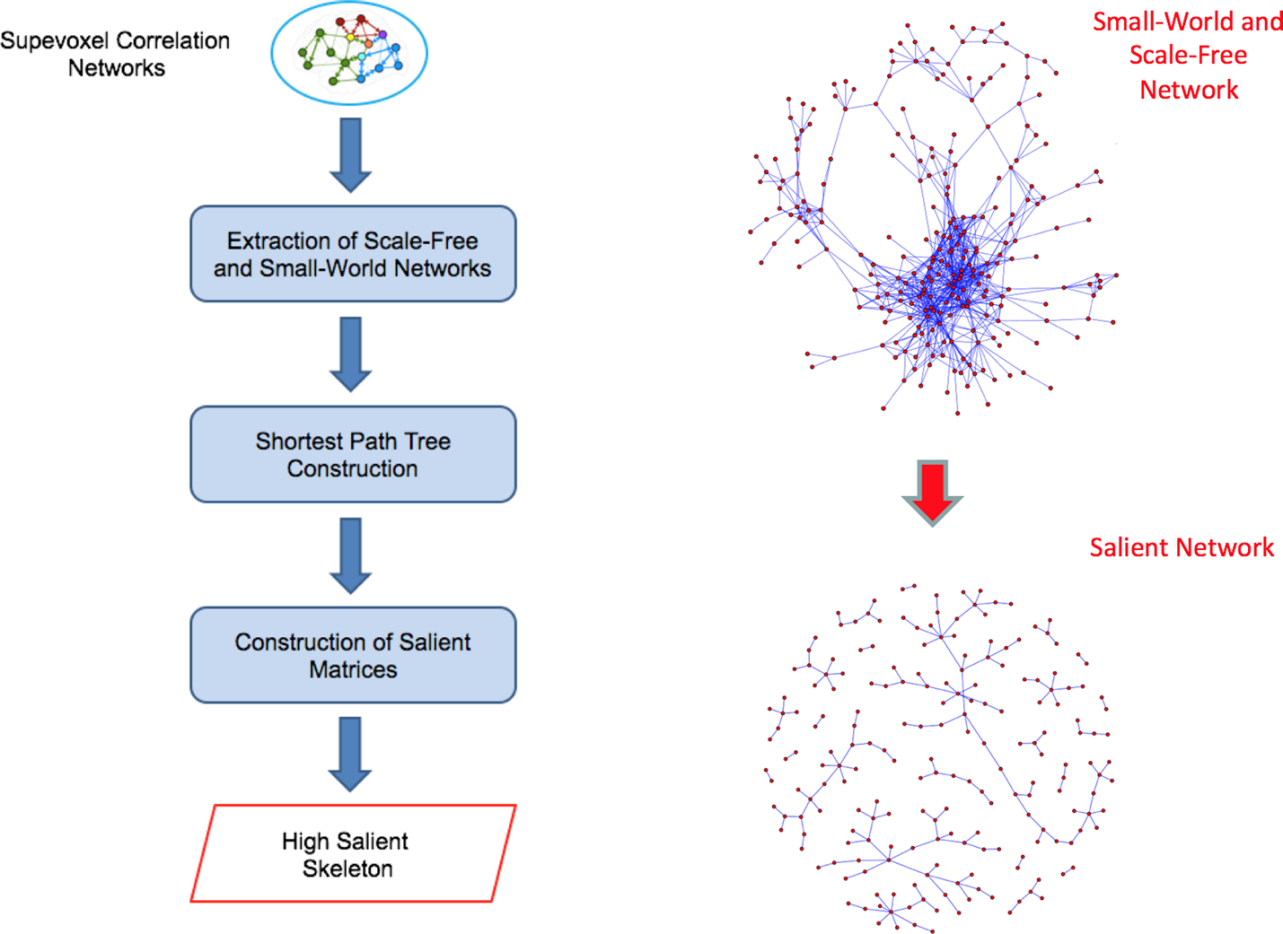
Flowchart of the salient skeleton construction. On the left the procedure to get the high salient skeletons is reported. It consists of different steps: scale free networks with power-law distributed weights were extracted from initial network of each subject, then links participating at least once in the shortest paths, starting from a fixed reference node, were recorded in the shortest path tree matrix, finally shortest path tree for each reference node were added up to obtain, for each subject, salience matrix, whose values $s_{i,j}=1$ represent high salient skeleton. On the right, a small-world and scale-free network is represented with the corresponding high salient network

The image processing and network construction were performed for each subject, thus a set of densely connected weighted networks was obtained. To reduce complex networks to their main components is an usual practice to examine a wide range of threshold values and then, according to specific experiment goals, choose the criterion for finding the most suitable one. A possible strategy to use for this investigation concerns the network topology which as disclosed in the *Background* should be small-world and scale-free especially for biological networks [27]. Therefore, we studied how the topology of each network changes when removing edges below a particular correlation value $r_{thr}$. To investigate network scale-free behavior, the fit goodness between the network degree distribution of each subject with a power-law in terms of adjusted R-squared statistics, was measured. While to study network small-world behavior, the small-worldness indicator of each subject network as a function of the threshold was computed. Small-worldness is given by the following expression:

1 $$\begin{aligned} SW=\frac{1}{M} \sum _{m=1}^M \left( \frac{C_m}{C_m^r} \Bigg / \frac{L_m}{L_m^r}\right) \end{aligned}$$

where $C_m$ and $L_m$, and $C_m^r$ and $L_m^r$ are the clustering coefficient and the average shortest path length respectively of each network *m* to examine, and the reference random graph with the same node number N and the same link probability *p* given by the ratio between mean degree $\bar{k}$ and *N*. Once found the appropriate threshold able to bring out the two main topologies, the high salience skeleton was extracted for each network in order to investigate which connections between are more relevant. Fig. 2 shows in detail the procedure adopted to get high salient skeletons from the supervoxel correlation networks along with an example of the scale-free and small-world network obtained and its relative skeleton.

The salience indicator was born to furnish an overall network description from a node-specific perspective and define a consensus among nodes on the importance of each edge within the network. Given the set of weights $W=\{w_1,w_2, ..., w_{N^\prime }\}$, with $N^\prime$ being the number of *N* nodes remaining after the extraction of the scale-free and small-word topologies from the network, the pairwise distance matrix *D* is defined. $D_{i,j}$ elements are simply the correlation reciprocal values. Therefore, nodes with greater weights, thus more correlated, are closer. After defining the distance between two nodes, it is also possible to introduce a path length definition: if two supervoxels *i* and *j* are connected through a path *p* consisting of *k* steps, being them the terminal nodes of *p*, the length of *p* is simply the weight sum of the edges belonging to it. Accordingly, it is possible to define several paths connecting *i* and *j*, we considered the so-called shortest paths that are the paths of which weight sum is maximum. It has to be noted that according to this definition, the shortest path uniqueness is not assured. Recording all links belonging at least one time to the shortest paths connecting a generic reference node *n* to all the remaining nodes of the network, we can define for each reference node what is generally called the shortest path tree *T*(*n*) a matrix which represents, in fact, the most effective routes linking the node *n* to the network. Its elements $T_{ij}(n)$ are mathematically defined in the following expression:

2 $$\begin{aligned} T(n)_{ij} = {\left\{ \begin{array}{ll} 1 &{} \text{ if } \text{ ij } \text{ link } \exists \text{ in } \text{ the } \text{ shortest } \text{ path } \text{ from } \text{ node } n = (1,..,N^\prime )\\ 0 &{} \text{ if } \text{ ij } \text{ link } \not \exists \text{ in } \text{ the } \text{ shortest } \text{ path } \text{ from } \text{ node } n = (1,..,N^\prime ) \end{array}\right. } \end{aligned}$$

One can calculate the shortest path trees for all the nodes of the network, then for a generic edge (*i*, *j*) connecting the nodes *i* and *j*, salience $s_{i,j}$ is the indicator accounting how many times the edge belongs to a shortest path tree in respect to the total number of shortest path trees. Therefore, salient matrix is given by the following equation:

3 $$\begin{aligned} S = \frac{1}{N^\prime } \sum _{n=1}^{N^\prime } T(n) \end{aligned}$$

**Fig. 3:**
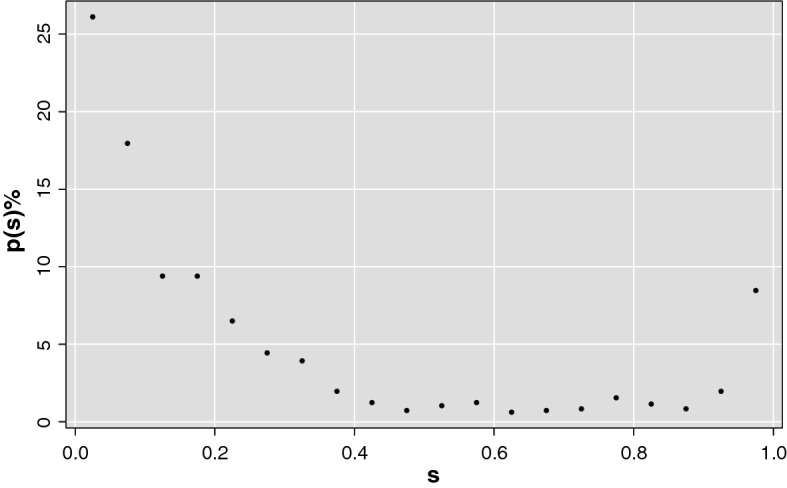
Bimodal distribution of the salient matrix values. The figure shows salience percentage frequency of a brain network as example. Saliency values are gathered on 0 and 1 thus it is possible to detect the salient links independently of the threshold value chosen. In this network the link fraction contributing to the high salient skeleton is of the $8.46 \%$

According to this definition, $s_{i,j}$ takes into account the fraction of shortest path trees including the edge between *i* and *j*. Salient matrix values have a characteristic bimodal distribution with peaks on 0 and 1 values, as shown in Fig. 3. If $s_{i,j}=1$, then link (*i*, *j*) is essential for all reference nodes, if $s_{i,j}=0$, (*i*, *j*) is not a fundamental link for the network. This makes natural finding what is called the salience skeleton, *i. e.* the backbone structure including all the most efficient links of the networks.

Figure 3 is an example of salience percentage frequency for a brain network. The salience skeleton is by definition the network containing only salient links. As the saliency distribution is bimodal there is no need of finding a particular threshold, but the skeleton stays pretty much unchanged for all threshold values between 0.1 and 0.9. For the present work the threshold 0.5 was used. It is worthwhile to notice that this bimodal behavior occurs independently of the clinical status.

### Supervised learning for the method evaluation

Each salient network was employed for building a skeleton multiplex network (SMN). For each layer, nodes are represented by the supervoxels that have a salient link at least in a subject and links, that change for each layer, are the absolute value of Pearson’s correlation between nodes pairs within the single layers.

From the SMN were extracted some centrality measures: the strength and the inverse partecipation ratio, first considering the single layer links, and then the multiplex network links. Multiplex network strength and inverse partecipation ratio were defined starting from an overall matrix *O* whose elements $o_{ij}$ indicate which links are present in at least a layer:

4 $$\begin{aligned} o_{ij}= {\left\{ \begin{array}{ll} 1, \quad \text{ if } \text{ link } \text{ ij } \text{ exists } \text{ in } \text{ at } \text{ least } \text{ a } \text{ layer } \\ 0, \quad \text{ otherwise } \end{array}\right. } \end{aligned}$$

As a consequence, for each node, strength and inverse participation ratio were weighted on the number of links incident upon a node over the whole multiplex network obtaining, in this way, two multiplex network quantities.

In addition, for this 4 quantities we computed the corresponding conditional means, thus strength and inverse participation averaged over the nodes with the same degree.

Overall we retrieved a matrix of 8N features × M samples, where N = 549 is the number of nodes of the SMN and M = 67 is the number of layers of the SMN.

To avoid over-training issues yielded by the excess of features compared to observations, a feature selection was performed with a wrapper-based method. The most important features were collected for each fivefold Random Forest cross-validation and then, those having a significant probability of occurrence within 1000 rounds, were selected. Random Forest was grown with 500 trees and the important features were chosen in order to overcome the third quartile of the importance distribution computed in terms of mean accuracy decrease.

The selected features were used to train another independent RF classifier always with 500 trees. Examples randomly selected to stay out of the training set were then adopted to measure the classification performance on the train subjects in order to evaluate the informative content of the available base of knowledge and detect which regions show an anomalous behavior. This is a useful information for those diseases whose patterns are not yet fully understood. Besides, a comparison of the performances obtained from the SMF, with those obtained from the original multiplex network and from their combination was carried out to deepen the characterization of the salient backbone of the human brain network in the field of the neurodegenerative diseases. Another characterization analysis concerns hub study of the salient network. As hubs are nodes with a number of links that greatly exceeds the average [28], we considered hubs all those nodes that are outliers of the network betweenness distribution. Therefore, for each subject, we collected nodes having a betweenness greater than the following quantity:

5 $$\begin{aligned} Q_3 + 1.5 * IQR \end{aligned}$$

where $Q_3$ and *IQR* are respectively the third quartile and the interquartile range of the betweenness distribution values of a generic network. On the base of this definition, hubs of the salient network and the original one were collected in order to see how many hubs are preserved moving from the original network to the salient one. In addition, for both the original network and the salient one we computed two metrics: strength and betweenness associated to the hubs of each subject to examine if these metrics were able to distinguish significantly (*p* < 0.01 using Wilcoxon Mann--Whitney test) the different clinical groups considered. An investigation of the anatomical region corresponding to the hubs that have a central role in pathological changes detection, was also carried out.

Another task, in this work, was the assessment of the methodology on the validation subject sets performing a binary classification: normal controls versus MCI converter subjects and normal controls versus Alzheimer’s disease subjects. For validation subjects, features were extracted adding in turn the test subject to the training SMN holding fixed the overall matrix. For each classifier, the tree number was set large enough to reach the typical training plateau for the out-of-bag error. At each split, being f the feature number, $\sqrt{(}f)$ features were randomly sampled.

The first task is certainly of interest for an assessment of the methodology and the feature informative power. In fact, it is well known which there are brain regions related to AD, thus we can use this classification task to observe whether or not high salience skeleton outlines brain regions coherent with the pathology. The second task is of paramount importance for clinical purposes, in fact especially the MCI condition can be in several cases a prodromal stage of AD. Accordingly, the early and accurate detection of impairment could play a pivotal role in the development of drug trials and therapy developments.

## Results

### Two main brain network topologies

**Fig. 4:**
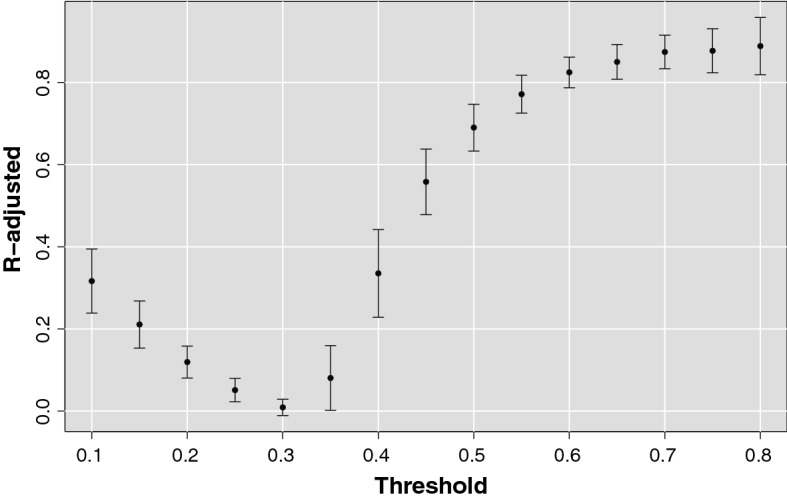
R-adjusted as a function of the threshold. The brain networks exhibit a power-law degree distribution for thresholds above 0.6. The goodness-of-fit is measured by means of adjusted R-squared coefficient. For each threshold value is reported the mean R-squared coefficient over all subjects and the relative standard deviation

There is not any in advance reason for which the scale-free and small-world topologies should have emerged with the same threshold value from different subjects. Nevertheless, Fig. 4 shows how the adjusted R-squared metric averaged over all subjects, adopted to measure the agreement between degree distributions and a power-law function, reaches a high and stable plateau for $r_{thr} > 0.6$

**Fig. 5:**
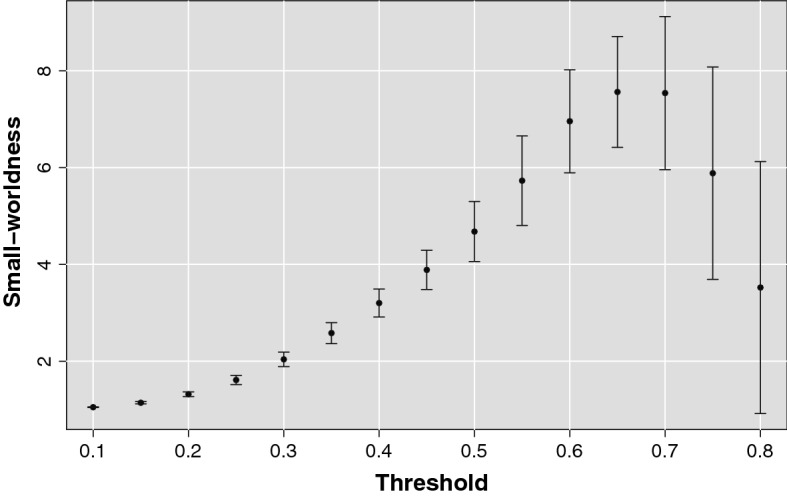
Small-worldness as a function of the threshold. The brain networks manifest an evident small-worldness behavior for thresholds above 0.6. For each threshold value is represented the mean small-worldness coefficient over all subjects and the relative standard deviation.

Moreover, considering the mean small-worldness indicator *SW* averaged over all subjects reported in Fig. 5 for each threshold value, it can be noticed as at a threshold above 0.6 in addition to a scale-free topology also a small-world structure emerges.

Coming back to the original brain network of each subject, we removed the correlations exceeding a threshold value of 0.65 chosen in order to bring out both significantly small-world and scale-free behavior of the networks minimizing variance of the R-adjusted and small-worldness indicators over the subjects. In this way, for each subject, a scale-free weighted network of highly correlated nodes was obtained.

### Assessment of salient skeleton methodology

First of all, we investigated the informative content of the high salient skeletons to evaluate if it exhibits advantages compared to the complete multiplex network. In the classification of NC versus AD patients, the important features, extracted from the skeleton multiplex network, gave performances, reported in terms of accuracy, area under the receiver-operating-characteristic curve (AUC) and its relative standard deviation, in keeping with the ones obtained using the original multiplex network. In fact, an AUC of $0.93 \pm 0.01$ and an accuracy of $0.85 \pm 0.01$ were found for the reduced multiplex network and an AUC of $0.94 \pm 0.01$ and an accuracy of $0.88 \pm 0.01$ were reached for the whole multiplex network. Corresponding specificities and sensitivities were respectively $0.83 \pm 0.01$ and $0.91 \pm 0.02$, for the first multiplex, and $0.85 \pm 0.09$ and $0.95 \pm 0.02$, for the second one.

**Fig. 6:**
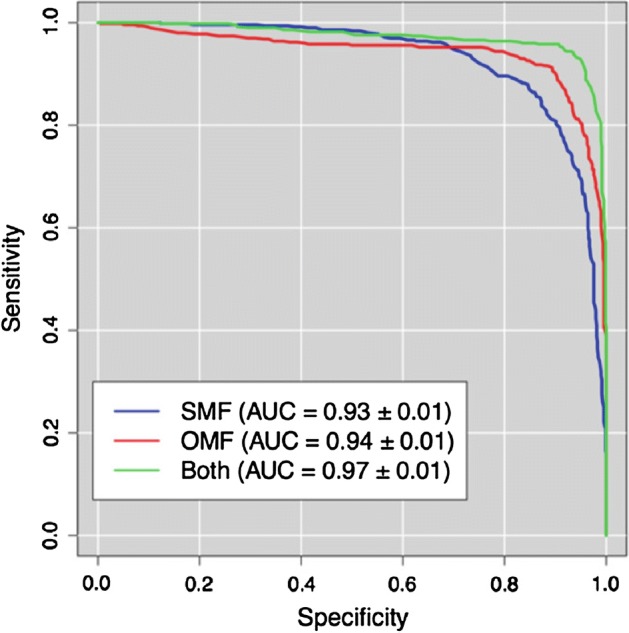
Skeleton evaluation with a receiver-operating-characteristic (ROC) curve comparison. In figure are reported, for the binary classification normal controls versus Alzheimer’s disease patients, the receiver-operating-characteristic (ROC) curves and the corresponding areas under the curve (AUC) relative to skeleton (blue curve), original (red curve) and both (green curve) multiplex network features

In addition, the significant features extracted using the salience approach were not the same obtained with the previous methodology. As a consequence, for assessing if the salient skeletons bring out different relevant information regarding AD disease pattern, the important features, extracted from the two approaches, were combined and used for distinguishing NC and AD. Receiver-operating-characteristic (ROC) curves for the three binary classifications NC versus AD, carried out respectively, using original (OMF), skeleton (SMF) and both multiplex features, were represented in Fig. 6.

This study showed that the feature combination makes it possible to achieve an higher classification performance with an AUC of $0.97 \pm 0.01$, an accuracy of $0.91 \pm 0.01$ and a specificity and sensitivity respectively of $0.88 \pm 0.06$ and $0.98 \pm 0.06$, demonstrating that the skeleton extraction from the network is able to provide additional important information within the multiplex framework.

### Hub characterization

**Fig. 7:**
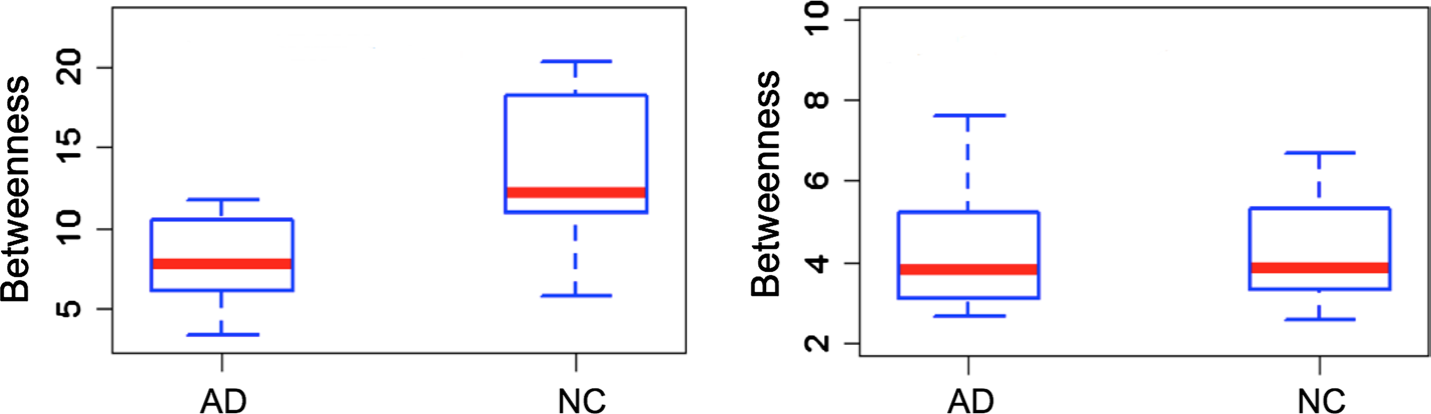
Betweenness distributions of an hub for the salient and the complete networks On the left, the boxplots of the betweenness distribution relative to an hippocampal hub of the salient networks are shown for healthy subjects and patients. On the right, the boxplots of the betweenness distribution corresponding to the same hub of the complete networks and for the same clinical groups are displayed. Hub associated to the salient networks allow us to distinguish AD and NC classes with a statistical significance level of $1 \%$, this does not occur for the original networks

Another adding value given by salient networks relates the study of the hubs. In fact, going from original network to the salient backbone, on average 70 % of the hubs is conserved. Besides, the salient network measures associated to the 15 hubs common to all subjects allowed us to statistically distinguish between NC versus AD and cMCI versus AD with a $1 \%$ significance level. On the contrary, complete network hubs are not able to reveal these statistical differences. In Fig. 7 an example of betweenness distribution relative to an hub covering a part of the left hippocampus is reported for both the salient skeleton and the original network using the boxplots of two different clinical groups (AD-NC).

**Fig. 8:**
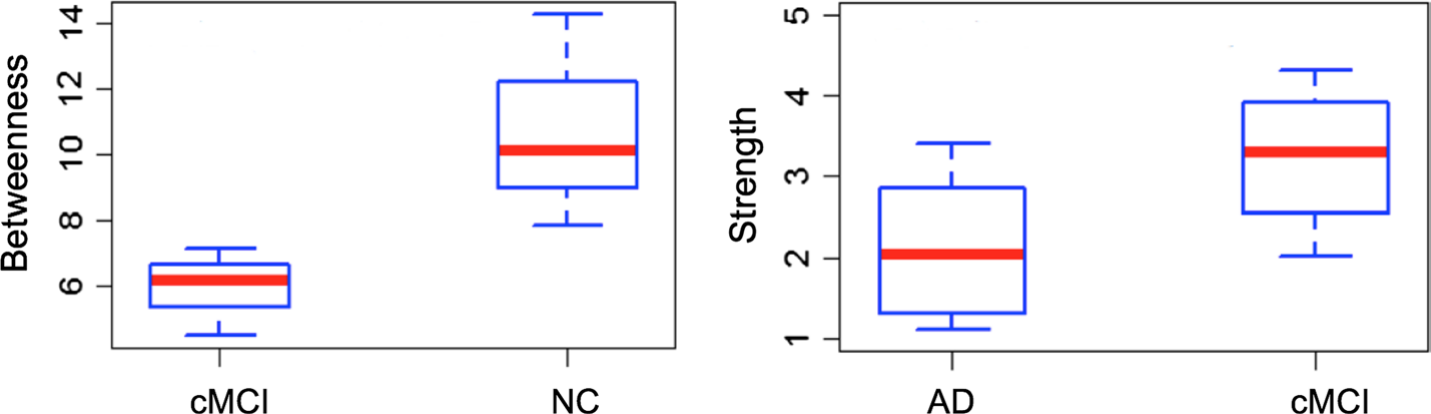
Examples of clinical group distinction through strength and betweenness distributions associated to two salient network hubs. On the left, the boxpltots of betweenness distribution of a salient network hub are represented for cMCI and NC classes. On the right, the boxplots of strength distribution of a second salient network hub are reported for AD and cMCI classes. Both of the network measures, associated to the two hubs, separate the clinical groups at a $1 \%$ significance level

In Fig. 8, instead, salient network betweenness and strength distributions associated to other two hubs are set out. It is possible to notice as in these two examples betweenness is able to statistically discriminate NC and cMCI, while strength statistically distinguishes cMCI and AD.

**Fig. 9:**
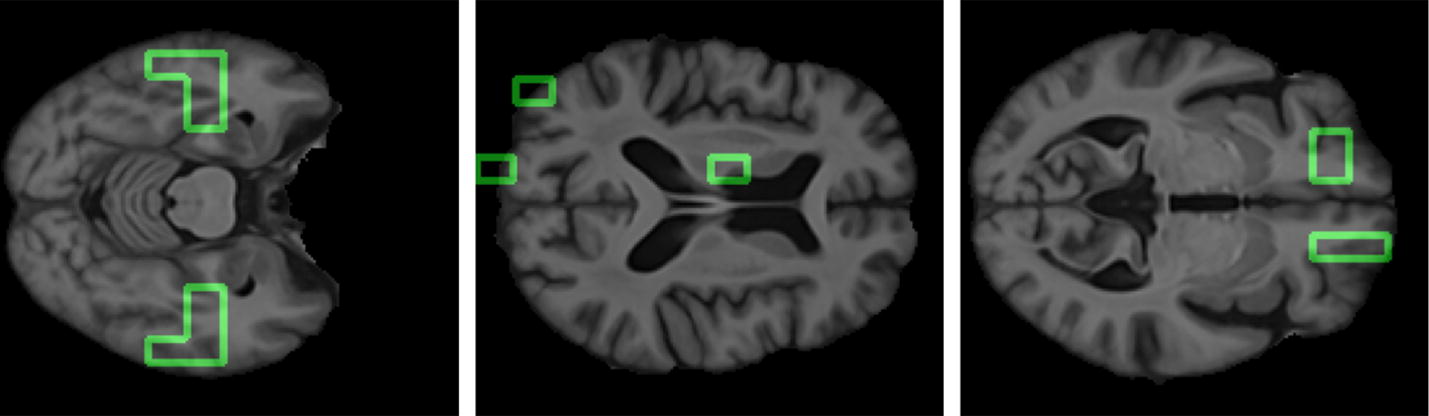
Anatomical hub visualization. The figure shows the supervoxels (green boxes) that are hubs and underline in the salient networks a connection with the clinical status. Hubs are represented along the axial planes of the template

In Fig. 9, the significant supervoxels, corresponding to the salient network hubs of which strength and betweenness are able to discriminate among different clinical groups, are represented on the axial planes of the brain template. It is worthwhile to consider that all these 15 hubs, in the complete networks, does not give any significant difference although they correspond to anatomical area importantly connected to AD.

### Salient skeleton reliability

To test the reliability of the skeleton multiplex network features (SMF), the classification models obtained during the training phase were used for the validation on the same dataset employed for validating the original networks, two binary classifications: controls vs. AD patients and controls vs. cMCI subjects were carried out.

The performances shown in Table 2, along with the ones obtained from the original multiplex network features (OMF), show as the method is reliable and as the most informative power of the complete multiplex network is retained by the skeleton multiplex network. In fact, salient networks allowed us to reduce by $92 \%$ the link number and by $82 \%$ the node number of the original networks giving accuracy lessening by up to $10 \%$. This is an important advantage especially when data scientists are working with data of great cardinality and high complexity (Big Data).

**Table 2:** Comparison of the classification performances of the salient multiplex network features (SMF), the original multiplex network features (OMF) and their combination (Both) in terms of accuracy, sensitivity specificity and the relative standard errors for the different groups: AD-NC and cMCI-NC

Metric	Feature Name	AD (48)-NC (52)	MCIc (48)-NC (52)
Accuracy	SMF	$0.79 \pm 0.01$	$0.76 \pm 0.02$
	OMF	$0.86 \pm 0.01$	$0.84 \pm 0.01$
	Both	$0.87 \pm 0.01$	$0.82 \pm 0.01$
Specificity	SMF	$0.81 \pm 0.02$	$0.82 \pm 0.02$
	OMF	$0.96 \pm 0.01$	$0.94 \pm 0.01$
	Both	$0.93 \pm 0.02$	$0.86 \pm 0.02$
Sensitivity	SMF	$0.77 \pm 0.02$	$0.71 \pm 0.02$
	OMF	$0.74 \pm 0.01$	$0.72 \pm 0.01$
	Both	$0.78 \pm 0.02$	$0.76 \pm 0.02$

In this regard, it is worthwhile to notice, the total amount of CPU time for the whole analysis pipeline based on the salient networks was of 58 h 42 min and 25 s. Therefore, each image processing and the relative subject prediction required a CPU time of 16 min 23 s and 2GB of RAM. This means that the reduction methodology of the salient skeletons provides a relevant computational saving, indeed the time taken to perform the whole analysis with the original networks is about the twice. It is important to specify that we used a single core 2.4 GHz CPU.

### Anatomical characterization

**Fig. 10:**
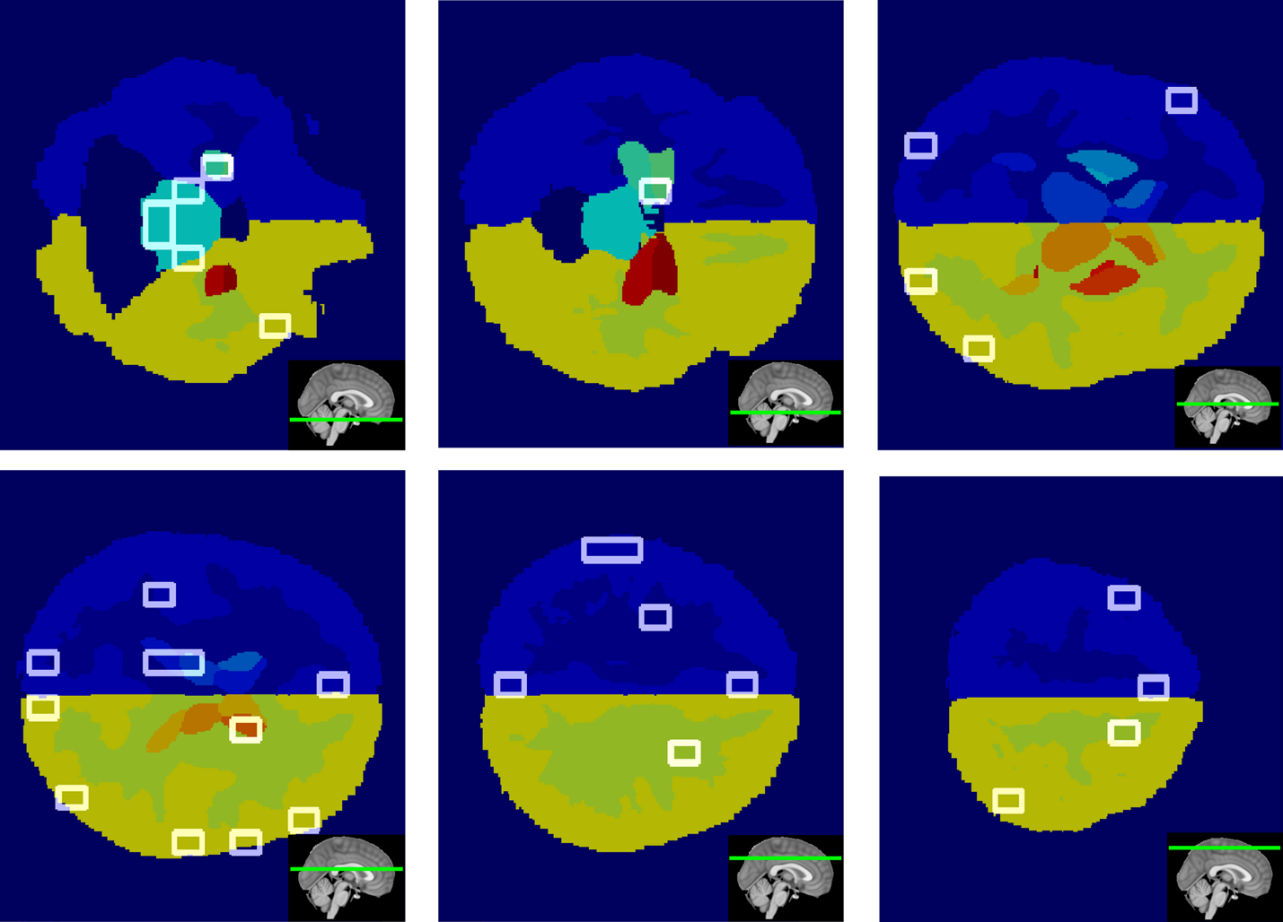
Important supervoxel visualization. The figure shows the supervoxels (white boxes), significantly connected with Alzheimer, along the different axial planes of the Harvard-Oxford atlas. The insets depicted in the bottom right corners represent position of each axial plane along sagittal plane

In this work, it was also studied where are located the significant supervoxels of the salient skeleton. Although the skeleton networks were completely different from the original dense networks and the important features selected were not the same, it is resulted that the significant supervoxels identified anatomical regions associated to the AD progression in keeping with the literature. In Fig. 10 the relevant supervoxels obtained on the axial planes of the Harvard-Oxford atlas with the relative axial plane position along sagittal view are represented.

It is worthwhile to note as significant supervoxels covered regions such as hippocampus, amygdala, ventricles, thalamus, brain stem, cerebral sulci, inter-hemispheric portions and separation areas between gray and white matter. These findings are a further confirm that salient skeletons are an alternative way of detecting AD patterns, able to give supplemental information regarding the disease.

## Discussion

The proposed approach aims at reducing dense and large brain networks preserving and underlining the meaningful information. In fact, nowadays especially in the neuroscience field, the necessity to perform multimodal longitudinal and even multi-scale studies has been increasing more and more and the management of large and numerous dataset has been becoming a really challenging task. Methodology includes two reduction steps. The first concerns the threshold study to extract the two fundamental topologies of the brain networks. This investigation provides two strategical aspects. On one hand, it confirms human structural brain networks analogously to the functional ones [29] can be described approximately as combination of a small-world and scale-free topology. On the other hand, all the brain correlation networks exhibit the general tendency to bring out these two topologies within a threshold intermediate range [0.6; 0.8]. This suggests that at too low thresholds, these structures are blurred by network noise while at too high thresholds, they begin to fail because link and node number becomes too scarce. The second reduction step consists in the use of the salience indicator to detect the most important links of the brain networks. Salient and original network features were evaluated and compared with a multiplex network approach. The analyses highlighted different advantages provided be salient skeletons: (i) the reduction of the computational burden keeping the fundamental information; (ii) accurate classification respectively of NC versus AD and NC versus cMCI subjects; (iii) identification of anatomical regions related to the disease; (iv) additional information compared to the complete networks. Indeed, as to the latter point, only network properties associated to the 15 salient network hubs are able to statistically distinguish NC, AD and cMCI. This becomes particularly interesting with a view of finding new biomarkers of disease progression that can be useful for the early diagnosis of neurodegenerative disease both in terms of discrimination accuracy and for the discovery of novel anatomical regions affected by the disease. It is worth to notice that mild cognitive impairment subjects represent a spurious class as there is no a priori clinical reason for this impairment to be related to AD instead of other neurodegenerative disease, this is why we tend to specifically focus the validation on cMCI subjects who do not have any clinical ambiguity. Therefore, cMCI can be accurately discriminated on the basis of the knowledge obtained from models constructed on NC and AD subjects [30]. Literature has a great number of articles dealing with the discrimination of NC vs. AD and NC vs. cMCI. However, the different data, processes, assessment and classification techniques used do not always allow a completely fair comparison. This is why it is important to organize International challenges [12, 31] to validate different methodologies with a common set of data and evaluation procedures. Apart the differences due to the previously enclosed reasons, in Table 3 it can be observed that our performances are comparable with the state-of-the-art [32, 33] although the optimization of the diagnostic accuracy was not the primary goal of this work.

**Table 3:** This table reports a comparison in terms of accuracy between our method and some of the most recent works regarding the study of the early AD diagnosis using MRI features

Salvatore et al. [34]	cMCI vs. NC (20-fold)	$0.72 \pm 0.12$
Salvatore et al. [35]	AD vs. NC (fivefold)	$0.90 \pm 0.05$
Lama et al. [36]	AD vs. NC (leave-one-out)	0.80
	AD vs. NC (tenfold)	0.77
Salvatore et al. [37]	AD vs. NC (fivefold) with MMSE	$0.96 \pm 0.01$
	cMCI vs. NC (fivefold)	$0.79 \pm 0.03$
Proposed method	AD vs. NC (fivefold)	$0.87 \pm 0.01$
	cMCI vs. NC (fivefold)	$0.82 \pm 0.05$

## Conclusions

In this article, we proposed a novel method for reducing complexity of dense and large brain networks, keeping and especially highlighting, their informative content. A threshold analysis was performed to bring out the two main topologies (small-world and scale-free) of the brain networks. In fact, the most of the behaviors of integration and segregation involved in brain organization are explained by these topologies. We also examined how a neurodegenerative disease, such as Alzheimer, can change structural connectivity of these brain networks extracting high salient skeleton for each one, in order to turn particular attention to the hubs and highways of these correlation networks which could be damaged by AD.

We assessed the informative power of the high salient networks through a multiplex network approach. Feature extracted from the skeleton multiplex network give in cross-validation, for the distinction of AD subjects from normal controls, an accuracy of $0.85 \pm 0.01$ which reaches $0.91 \pm 0.01$ combining these features with those obtained using the original multiplex network. The improvement of the classification performances and the different features extracted from the skeletons prove that salient links underline an information, at least in part, distinct from that obtained with the initial networks. This was also confirmed by the hub study. Indeed, even though $70 \%$ of the original network hubs are preserved, a comparison among the same hubs of the salient skeletons and the complete networks demonstrated that only the network metrics associated to the hubs of the salient networks allow us to distinguish the three clinical groups with a $1\%$ significance level. It was also showed as these hubs, anatomically, pinpoint brain regions related to AD. Therefore, having a method able to underline the relevant information of these structures is fundamental. Methodology importance was also emphasized by the study of the anatomical regions selected by the important classification supervoxels corresponding to areas, such as hippocampus, notably connected with Alzheimer disease progression. In addition, on an independent set of subjects, the methodology was validated performing two binary classifications: NC versus AD subjects and NC versus cMCI subjects. Results demonstrate the reliability of the salient network approach that permits us to reduce significantly the original networks ($92 \%$ of the links and $82 \%$ of the nodes) keeping high performances that undergo a reduction smaller than $10 \%$. It is important to notice this performance reduction is negligible if compared to the time saved for the whole analyses that would be lasted twice as long using the complete networks. Methodology here developed can have application in the study of other neurodegenerative diseases and in all fields where, managing a great quantity of data and large complex networks in a way computationally convenient, and revealing network characteristics that otherwise would remain masked, can be really important. Further in-depth to this study could be the investigation into other network properties of the salient networks and an examination on a larger dataset of MCI subjects. In addition, a study about hub vulnerability in AD and NC salient networks could be really interesting.
